# Techno-Functional Properties of Burgers Fortified by Wild Garlic Extract: A Reconsideration

**DOI:** 10.3390/foods12112100

**Published:** 2023-05-23

**Authors:** Vladimir S. Kurćubić, Slaviša B. Stajić, Nemanja M. Miletić, Marko M. Petković, Marko P. Dmitrić, Vesna M. Đurović, Volker Heinz, Igor B. Tomasevic

**Affiliations:** 1 Department of Food Technology, Faculty of Agronomy, University of Kragujevac, Cara Dušana 34, 32000 Čačak, Serbia; 2 Department of Animal Source Food Technology, Faculty of Agriculture, University of Belgrade, Nemanjina 6, 11080 Belgrade, Serbia; 3 Veterinary Specialist Institute Kraljevo, Žička 34, 36000 Kraljevo, Serbia; 4 Department of Microbiology and Microbiological Biotechnology, Faculty of Agronomy, University of Kragujevac, Cara Dušana 34, 32000 Čačak, Serbia; 5 DIL German Institute of Food Technology, Prof.-von-Klitzing-Str. 7, D-49610 Quakenbrück, Germany

**Keywords:** wild garlic, freshly squeezed extracts, burger, antioxidant, antimicrobial, technological properties, sensory quality

## Abstract

The aim of this research was to examine the chemical properties of freshly squeezed wild garlic extract (FSWGE) and its use as an additive in burgers (BU). Technological and sensory properties of such fortified burgers (BU) were determined. LC-MS/MS analyses identified thirty-eight volatile BAC. Allicin prevalence (11.375 mg/mL) is the key parameter determining the amount of FSWGE added in raw BU (PS-I 1.32 mL/kg, PS-II 4.40 mL/kg, and PS-III 8.79 mL/kg). Minimum inhibitory concentrations (MIC) and minimum bactericidal concentration (MBC) values of the FSWGE and evaporated FSWGE (EWGE) were determined against the six microorganisms using a microdilution method. The data indicated that using FSWGE can result in a reduced risk of *Serratia marcescens* (MIC = 50 mg/mL; MBC = 60 mg/mL), *Listeria monocytogenes* (MIC = MBC = 90 mg/mL), *Escherichia coli* and *Staphylococcus aureus* (MIC = 90 mg/mL; MBC ≥ 100 mg/mL), and *Salmonella enteritidis* and *Enterococcus faecium* (MIC = 100 mg/mL; MBC > 100 mg/mL) in BU. Changes in antioxidant (AOX) capacity were followed during cold storage (up to 10 days) and freezing (90 days). It was shown that PS-III had the highest level of AOX capacity during the entire period of cold storage, revealing 8.79 mL FSWGE/kg BU as the most suitable effective concentration. Adding FSWGE did not negatively affect the technological and physico-chemical properties during both cold and freeze storage. Regarding sensory evaluation, modified BU received mostly higher scores compared to control. The results of this study have demonstrated the great potential of wild garlic extract usage in the creation of safe products with prolonged shelf life.

## 1. Introduction

Burgers are a very popular meat product consumed all over the world, especially by younger consumers. The diet in which burgers (BU) are often represented (as the so-called “fast” or “junk” food) is considered to be of poor quality and risky for the health of consumers [1,2,3]. The main harmful effects of consuming such food are increased childhood and adult overweight/obesity, cardiometabolic risk, high blood pressure, and dental caries [3,4,5,6,7]. The use of herbal extracts can improve food quality and nutritional value, which is why incorporating medicinal and spicy herbs and their extracts into innovative food and functional products are especially relevant [8,9]. Significantly improved consumer awareness of buying and consuming healthier meat products with desirable nutritional characteristics has led to a series of studies crowned with the reformulation of certain traditional products [10,11,12,13,14]. Consumers believe that innovations in traditional product formulations must not lead to the degradation of their sensory properties [15]. Thus, enriching meat products using herbal extracts as a preservative or an additive and adding natural healthy ingredients with the aim of developing new functional foods with desirable technological properties can be a winning strategy [16,17,18,19,20,21,22].

*Allium ursinum* L. is a member of the Amaryllidaceae family. It is called European wild garlic (WG), with several synonyms: ramson, wood garlic, or bear’s garlic. WG is a perennial plant species widespread throughout Europe and Asia [23]. “The new star” of garlic and European “Medicinal Plant of the Year” are flattering titles that WG has received for the significant activities it exhibits [24]. WG is often used in traditional medicine and gastronomy without side effects on the environment [25]. The results of scientific studies confirm the indications for the effective use of WG in the prevention/therapy of a large number of different diseases: it significantly lowers blood pressure, cholesterol, and triglyceride levels in the serum, which prevents the occurrence of diseases of the cardiovascular system [26,27,28,29,30,31]; it has antimicrobial effects [23]; it protects against cold and bronchitis [32]; and it facilitates wound healing and treatment of chronic skin diseases [27]. The beneficial activities of WG on human health can be attributed mainly to the sulfurous compounds, which are most abundant in the *Allium* species. Very important chemical constituents of WG leaves are polyphenols (ferulic and vanillic acid, p-coumaric acid, and kaempferol derivatives) and high concentrations of flavonoids [33,34,35]. WG leaves also contain chlorophylls and carotenoids, vitamin C, and microelements. Moreover, the iron content of 247.9 mg/kg is not negligible [36,37]. The presence of the mentioned valuable bioactive compounds makes it suitable for creating innovative healthier/functional foods.

Modern consumers prefer to prepare food simply and quickly. Researchers, scientists, and the meat industry are synergistically developing modified “instant” or “fast food” products that offer health benefits to consumers, with synthetic substances replaced by natural ones [38]. Our research is innovative and comprehensive: it includes the precise chemical characterization of WGE and the selection of the most effective concentration as a preservative, antioxidant, and spice. Determining total phenolic content and antioxidant capacity in the complex matrix of conventional and experimental modified BUs (1, 5, and 10 d of cold storage and on day 90 of freeze storage) is particularly significant for their shelf life. About 100 billion BUs are sold annually worldwide [39]; hence we considered it extremely important that our study should examine their technological properties (cooking loss, raw and grilled diameter), physicochemical parameters (pH, moisture, protein content, and fats), and sensory acceptance.

## 2. Materials and Methods

### 2.1. Plant Material

The leaves of *Allium ursinum* L. were collected in Central Serbia in March 2020, in the lower part of Mt Ovčar, a protected area of exceptional features (The Ovčar-Kablar Gorge, latitude 43°54′02.8″, longitude 20°11′54.7″ and 391 m above sea), at its pre-flowering stage. Fresh plant leaves were hand selected for freshly squeezed extract preparation. This approach is justified because there is a risk that due to exposure to high temperatures, oxygen, and light (to which they are sensitive), certain bioactive compounds may degrade in the WGE. The species was identified, and the voucher specimen was deposited in the Herbarium of the Institute of Botany and Botanical Garden “Jevremovac,” University of Belgrade–number 17817 BEOU [40].

### 2.2. Preparation of Freshly Squeezed Wild Garlic Extract

Freshly squeezed wild garlic extract (FSWGE) was prepared by cold-pressing chopped leaves in a manual squeezer (garlic press BL-3455, Blaumann, Budapest, Hungary). Squeezing was repeated several times to obtain approximately 300 mL of representative extract.

### 2.3. LC-MS/MS Analyses

FSWGE was analyzed using the Waters liquid chromatographic system Acquity H Class consisting of quaternary pump Acq QSM, and autosampler Acq FTN with column heater connected to a Xevo TQD mass spectrometer (Waters, Milford, CT, USA). Separation was carried out on a reverse phase Acquity BEH column (I.D. 2.1 mm × 150 mm, 1.8 μm particle size, Waters). The flow rate of the mobile phase was 0.4 mL/min, while the injection volume was 5 μL. The column oven was set at 40 °C. Eluents A and B were H_2_O and acetonitrile (ACN), respectively. Both eluents contained 0.1% formic acid and NH4OH. Gradient elution was performed by changing the mobile phase composition as follows: 0 to 10 min, 95% A, to 5% A using a linear gradient.

All samples were diluted 10 times with water and filtered through 0.22 μm microporous membrane before LC-MS/MS analysis. An MS system equipped with an electrospray ionization source (ESI) was operated in MRM and positive- or negative-ion scan mode at the same time. ESI ionization conditions were as follows: capillary voltage = 3.5 kV, cone voltage = 30.0 V, source temperature = 140 °C, desolvation temperature = 350 °C, desolvation gas flow = 550 L/hr, and cone gas flow = 50 L/hr. MassLynx 4.1 software (Waters, Milford, CT, USA) was used for data acquisition and processing.

Calibration curves were obtained from standard solutions at different concentration levels, selected as representatives of the range of concentration in the sample. The regression analysis of various concentrations of standard solutions (0.08–8 µg/mL) gave good correlation coefficients for the calibration curves of allicin (R^2^ = 0.9974), ferulic acid (R^2^ = 0.9916), p-coumaric acid (R^2^ = 0.9957), and sinapic acid (R^2^ = 0.9981). Allicin standard was purchased from Allicin International (East Sussex, UK), with a purity of ≥98.0%, while standards of phenolic acids were purchased from Sigma-Aldrich (Burlington, MA, USA), with >99% purity.

### 2.4. Sample Preparation for Qualitative Analysis

Essential oils from the *Allium ursinum* L. (Wild Garlic) plant were extracted using Clevenger apparatuses. In the Clevenger apparatus, 180 g of sample mixed water is boiled to evaporate volatile components. Concurrently, in the steam distillation approach, steam is passed through a bed of the non-polar solvent, which is suitable for GC MS analysis, hexane in this case. The time to the finish destination is 2 h. Solvents with essential oils were transferred into a 4 mL vial, evaporated in nitrogen stream to dryness, and then reconstituted with 2 mL of hexane.

### 2.5. GC-MS Analyses

GC-MS analysis of *Allium ursinum* L. oils was carried out on Agilent 6890 GC (Agilent Technologies, Santa Clara, CA, USA), Agilent 5973 mass selective detector (Agilent Technologies), EI mode (70 eV), and 40–600 mass scan, and operated through an Agilent ChemStation data system (Agilent Technologies), HP-5 ms capillary column (30 m length × 0.25 mm inner diameter × 0.25 µm film thickness), helium carrier gas, head pressure (20,1 psi), flow rate (1.75 mL/min), and oven temperature program (60 °C initial temperature, which was increased at a rate of 3 °C/min up to 300 °C), with a run time 90 min, inlet temperature (300 °C), and interface temperature (280 °C). Injection volumes (1 µL) were injected using a splitless mode (100:1). The components of each essential oil sample were identified based on their retention indices and mass spectral fragmentation patterns compared to reference literature and our in-house library.

### 2.6. Antibacterial Activity, Minimum Inhibitory Concentrations and Minimum Bactericidal Concentration Values

The antibacterial (AB) activity was tested against the *Staphylococcus aureus* ATCC 25923, *Escherichia coli* WDCM 00013, *Listeria monocytogenes* ATCC 13932, *Salmonella enteritidis* ATCC 13076, *Enterococcus faecium* ATCC 6057, and *Bacillus subtilis* ATCC 6633 (in FSWGE) or *Serratia marcescens* ATCC 43862 (in EWGE). Bacterial colonies from the plates incubated overnight at 37 °C were resuspended in sterile NaCl and adjusted to the 0.5 McFarland standard. The inoculum prepared above was diluted at 1 × 10^6^ CFU/mL. Samples for examination were freshly squeezed WGE (FSWGE) and evaporated FSWGE (EWGE). Both extracts were filtered through 0.45 µm membrane filters (FiltropurS 0.45, Lot 90245103, Sterile, SARSTED AG & Co. KG, Sarstedstr. 1, D-51588 Nümbrecht, Germany). FSWGEs were used in the test at concentrations of 100%, 90%, 80%, 70%, 60%, 50%, 40%, 30%, 20%, and 10% (diluted in MHB). FSWGE (50 mL) was evaporated in an N_2_ atmosphere (Nitrogen Generator, MICRO, Tremezzina, Italy), maintaining a temperature of 40 °C in a water bath, in order to obtain dry WGE. An orange gelatinous EWGE was obtained. The initial concentration of 100 mg/mL of EWGE was dissolved in 10% DMSO. From EWGE concentrations of 100 mg/mL by dilution in sterile water, EWGE concentrations of 90, 80, 70, 60, 50, 40, 30, 20, and 10 mg/mL were prepared, respectively. Determination of minimum inhibitory concentrations (MIC) was performed following the European Committee on Antimicrobial Susceptibility Testing (EUCAST) guidelines using the broth dilution method in a 96-well microtiter plate with U bottom. In the microtiter plate, 50 µL of FSWGE (decreasing concentrations of 100%, 90%, 80%, 70%, 60%, 50%, 40%, 30%, 20%, and 10%) or EWGE (100, 90, 80, 70, 60, 50, 40, 30, 20, and 10 mg/mL) and aliquot of 50 µL of bacterial suspension was inoculated to each well (from the first to the tenth well). Fifty μL of Mueller–Hinton broth (MHB) was added to all wells. Both extracts were tested in 2 replicates (2 rows on a microtiter plate, 10 wells in the first, and 10 in the second row). In both rows, the eleventh well was a negative control (MHB only, sterility control); in the twelfth well, there was a positive control (MHB + bacterial inoculum). All samples were tested in duplicate, and the tests were repeated twice. Plates were wrapped loosely with original covers to ensure that bacteria did not become dehydrated and then placed in an incubator at 35 ± 2 °C for 20 h. Thirty μL of resazurin (TCI, Tokyo, Japan) indicator solution (prepared by 0.015% dissolution in sterile distilled water) was added to each well. After further incubation of 2–4 h, the color change was assessed visually. MIC was defined as the lowest concentration, in which no color change of resazurin was observed. To determine the minimal bactericidal concentration (MBC), 10 μL of well content was inoculated onto the MHB plate and incubated for 24 h. The lowest concentration that showed no growth after 24 h was taken as the MBC value.

### 2.7. Burger Preparation

The burgers (BU) were prepared in a small-scale plant Suvobor coop Ltd. (Čačak, Serbia), according to traditional routine procedures, on the same day (and in an identical manner). Four production series (PS) of BU (control—CON and three experimental—PS-I, PS-II, and PS-III), about 4.8 kg each, were prepared according to the following procedure: All formulations of BU were made with 50% pork shoulder, 20% beef shoulder, 15% fatty beef trimmings with 30% fat, 10% water, 2% table salt, 0.5% white pepper, 0.8% sweet red ground pepper, and 1% “Pergeta” cooking supplement with vegetables (Meat & Trade, Primorska 84C, Novi Sad, Serbia). Garlic powder (0.2%) purchased from Meat & Trade (Novi Sad, Serbia) was added only to the control. Frozen meat (stored at −20 °C for four weeks (pork) and five weeks (beef and beef trimmings, before use) was thawed in the cooling chamber at +4 °C for 10 h, until 0–2 °C of meat was reached. The meat was ground using a 14 mm plate (Grinder fi 114, Krämer + Grebe GmbH, Biedenkopf, Germany), then salted and aged for a minimum of 12 h. Additives and spices were added during mixing in a blender (Fimar—Villa Verucchio—Rimini—Italy, Model IC50CIP40050T, serial No. 130900591, 40 V, 1500 W, 50 Hz, IPX3, 2013) for 5 min. The second grind was to a diameter of 5.8 mm. FSWGE was added during mixing directly into minced meat in the following concentrations:

- CON = 0;
- PS-I = 15 ppm = 15 mg allicin/kg of BU = 1.32 mL FSWGE/kg of BU;
- PS-II = 50 ppm = 50 mg allicin/kg of BU = 4.40 mL FSWGE/kg of BU;
- PS-III = 100 ppm = 100 mg allicin/kg of BU = 8.79 mL FSWGE/kg of BU.

About 120 g of minced meat was weighed for each burger and shaped in a manual molding press (Hamburger Patty Molding Press with Handle, Garde HDHP34 Heavy-Duty 3/4 lb. 6″, Webstaurant Store, LLC, Lancaster, PA, USA). Burgers (9–12 BU in one food container) were packed in disposable polystyrene packaging (4 mm thick, BOXPAC DMD, Čačak, Serbia) according to Good Hygiene Practice, with clearly labeled sample groups for identifying samples. The whole process was repeated three times (three independent batches), and samples (at least three BUs per treatment and per test period) were stored at +4 °C (cold storage) for 10 days and at −20 °C (freeze storage) for 90 days. Analyses were performed on days 1, 5, and 10 during cold storage and after 90 days of freeze storage. Heat treatment was carried out in combi ovens (RATIONAL AG, Igliner Str. 62, D-86899 Landsberg am Lech, Mod. SCC WE 101, serial-no. E 11SI17112630902, Germany). The BUs were cooked at a set/programmed temperature of 285 °C for 9 min. Dedicated combi ovens allow for the thermal processing of foods of different flavors (e.g., fish and pork) in the same chamber without affecting each other. The uniformity of the action of high temperature on BUs has been achieved (by controlling the devices` thermometer and by measuring the temperature of samples of all treatments with a probe thermometer immediately after heat treatment), which is important for the validity of the sensory evaluation.

### 2.8. Determination of Total Phenolics and Antioxidant Capacity in Raw Burgers

Firstly, the alcoholic extracts of raw burgers were prepared on days 1, 5, and 10 for cold-stored BUs and day 90 for freeze-stored burgers. Four grams of raw BUs of each sample of all production series were mixed with 40 mL of 96% ethanol and ultrasonicated for 30 min. The extracts were centrifuged twice for 10 min at 3500 rpm, and the supernatant was filtered through a 0.45 mm Minisart filter prior to analysis. Utilizing a modified Folin–Ciocalteau colorimetric method [41,42] with results presented as mg of gallic acid equivalents/100 g raw BU (mg GAE/100 g mm) and an ABTS assay [43] with results presented as mmol Trolox equivalents/100 g raw BU, respectively, the total phenolic content and antioxidant (AOX) capacity were determined in such obtained extracts. The extraction was repeated three times (per treatment and per time period) in order to perform antioxidant measurements in triplicate.

### 2.9. Determination of Technological Properties and Proximate Composition

Four burgers from each treatment on days 1, 5, 10, and 90 were used to examine technological properties and proximate chemical composition.

Raw and cooked burgers were weighed on the scales with a 0.1 g precision ratio to determine cooking loss:

(1) $$\mathrm{cooking}\;\mathrm{loss}\;\left(\%\right)=\frac{{\mathrm{weight}}_{\mathrm{raw}}-{\;\mathrm{weight}}_{\mathrm{cooked}}}{{\mathrm{weight}}_{\mathrm{raw}}}\times 100$$

The diameter of each burger (raw and cooked) was measured in triplicate (horizontal, vertical, and at 45°angle), and calculated average values were used to determine diameter reduction (DR) using the following formula:

(2) $$diameter\;reduction\;\left(\%\right)=\frac{avg.\;diameter_{raw}-avg.\;diameter_{cooked}}{avg.\;diameter_{raw}}\times 100$$

pH values were measured twice on each burger (raw and cooked) with pH-meter Testo 206 pH2 (Testo AG, Lenzkirich, Germany) equipped with a penetration probe. Before each measurement, the pH meter was calibrated with standard buffer solutions (pH = 4 and pH = 7).

Moisture content was determined by drying samples at 105 °C [44]. Protein content was determined by the Kjeldahl method and multiplying by factor 6.25 [45]. Fat content was determined by the Soxhlet method [46]. These analyses have been conducted on grilled burgers.

### 2.10. Sensory Evaluation

**Figure 1:**
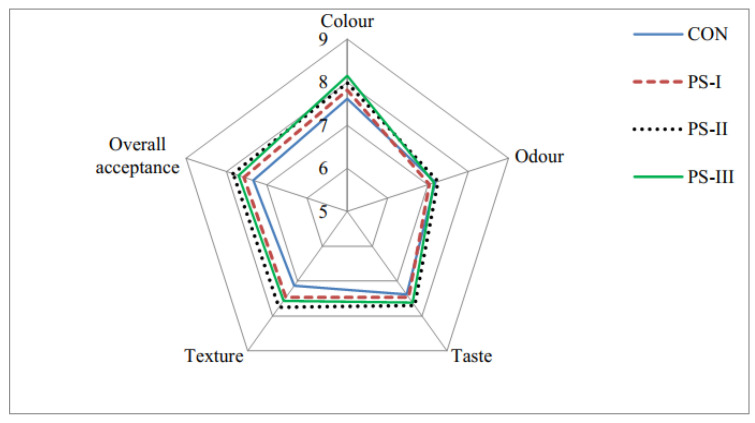
Results of sensory evaluation. CON—without FSWGE; PS-I with 1.32 mL of FSWGE/kg of BU; PS-II with 4.40 mL of FSWGE/kg of BU; PS-III with 8.79 mL of FSWGE/kg of BU; FSWGE—freshly squeezed wild garlic extracts; BU—burger.

Sixty consumers (aged 19–62, 61.67% male, 38.33% female) participated in the survey. They were selected on the grounds of consuming burgers (BUs) at least once a week. All consumers were chosen from among students and staff members of the Faculty of Agronomy, University of Kragujevac. Sensory analysis was performed simultaneously in the canteen of the Student Center in Čačak. After heat treatment, BUs of all treatments were cut into six pieces, samples were randomly coded with three-digit numbers and randomly served (one per consumer) on white plastic plates under natural daylight. Consumers were asked to evaluate the color, odor, taste, texture, and overall acceptability using a numeric-descriptive scale with a nine-point system (1—extremely unacceptable, 9—extremely acceptable). They used water at room temperature to cleanse their palate between samples. The survey was performed in a single testing session (day 1). The results are presented in Figure 1 as a radar-style chart generated in MS Excel 2016.

### 2.11. Statistical Analysis

The results were subjected to two-way ANOVA to evaluate the effect of WGE addition and storage time as fixed effects and their interaction with replicates as a random term. Additionally, the sensory analysis and proximate composition results were subjected to one-way ANOVA, considering treatments as a fixed effect, and the panelists and replicates, respectively, as a random effect. Analyses were performed by software Statistica 12.5 (StatSoft, Inc., Tulsa, OK, USA) and presented as a mean ± standard deviation (SD). Differences between means were determined using Tukey’s HSD test at the significance level *p* < 0.05.

## 3. Results and Discussion

### 3.1. Characteristics of WG Extracts

Using the LC technique, four compounds were detected and quantified in FSWGE (Table 1). The concentration of allicin, which is mainly responsible for the garlic aroma and most of the functional effects, was 11.375 mg/mL, and this value was the key parameter that determined the amount of FSWGE added to raw BUs in three different concentrations (PS-I: 1.32 mL FSWGE/kg BU; PS-II: 4.40 mL FSWGE/kg BU; PS-III: 8.79 mL FSWGE/kg BU). Organosulfur compounds are secondary plant metabolites (e.g., thiosulfates), biosynthesized as defense compounds against abiotic stressors that lead to plant tissue damage [47,48]. Other research studies also found that all three detected hydroxycinnamic acids (ferulic acid, p-coumaric acid, and sinapic acid) in WGE [26,49,50].

The use of WGE as an antioxidant and preservative for human food of animal origin is possible due to its low toxicity [27,30,51].

Volatile components of WG oils were investigated by the GC/MS analyses (Table 2). In total, 38 volatile compounds were identified: 27 organosulfur compounds (17 aliphatics, 8 heterocyclics, and 2 aromatic compounds), two alkanes (compounds 24 and 36), two aldehydes (26, 38), two chlorine-containing aromatic compounds (6, 21), nitrogen- and chlorine-containing aromatic compound (27), nitrogen-containing heterocyclic compound (12, 31), boron-containing heterocyclic compound (25), and unsaturated fatty acid ester (33). Nine compounds were unidentified (RT, min = 5.762; 11.248; 11.577; 61.907; 66.998; 70.350; 71.794; 75.044; and 80.302). Based on peak areas, the content of organosulfur compounds was 82.56%, while the oxygenated compounds made up 6.82% (Table 2).

All identified sulfides (28, 30), disulfides (2, 4, 8, 13, 15), trisulfides (1, 3, 5, 9), tetra sulfides (11, 23), pentasulfide (29), and dithiins (14, 16) in the WG were previously reported [25,30,52,53,54]. The most abundant volatile compound was methyl 2-propenyl trisulfide (21.63%), followed by diallyl disulfide (13.30%) and diallyl trisulfide (12.87%). A variation in the content and abundance of the most volatile compounds in WG oils in previous publications might be due to environmental conditions, harvest time, methods of oil preparation, etc. [54,55,56].

### 3.2. Antibacterial Activity

In order to perform a successful application of a naturally occurring antimicrobial to food, its efficacy needs to be determined. The initial screening revealed that FSWGEs exhibited powerful antibacterial activity (ABA) against all tested organisms, and more detailed studies of the ABA of EWGE, with a variable degree of their sensitivity. We consider the manifested ABA all the more significant because a very strong bacterial inoculum was used in this study (1 × 10^6^ CFU/mL). The results of ABA obtained by the dilution method are given in Table 3 and Table 4.

Minimum inhibitory concentrations (MIC) were determined for six bacterial strains. FSWGE exhibited excellent efficacy and the strongest ABA against *E. coli* (MIC = 20%; MBC = 30%), *S. aureus, S. enteritidis, B. subtilis* (MIC = 30%; MBC = 40%), and *L. monocytogenes* (MIC = 30%; MBC > 50%), and was somewhat less effective on *E. faecium* (MIC = 50%; MBC > 60%). According to the results, FSWGE shows great application potential as a food preservative. The highest susceptibility to EWGE among the bacteria tested was exhibited by *S. marcescens* (MIC = 50 mg/mL; MBC = 60 mg/mL), *L. monocytogenes* (MIC = MBC = 90 mg/mL), *E. coli* (MIC = 90 mg/mL; MBC = 100 mg/mL), and *S. aureus* (MIC = 90 mg/mL; MBC > 100 mg/mL). *S. enteritidis* and *E. faecium* have been shown to be the most resistant to the ABA of EWGE (MIC = 100 mg/mL; MBC > 100 mg/mL), but we believe that EWGE also showed a very strong ABA against them. The most important tested activity for the preservation of meat and meat products is the ABA against *L. monocytogenes, S. enteritidis, E. coli*, and *S. aureus* [57]. In fact, we were given the possibility of adding WGE to various foods in the amount of the obtained MIC values. It is also important to note that the MBC values of FSWGE and EWGE against most of the examined bacteria were slightly higher than the corresponding MIC values. The MIC and MBC values were equal in the case of EWGE influence against *L. monocytogenes*. The MIC test demonstrates the lowest level of ABA that is bacteriostatic (prevents the visible growth of bacteria), and the MBC demonstrates the lowest level of herbal ABA required to kill a particular bacterium. Our tests show that there are no major differences between specific concentrations of plant extracts that cause MIC and complementary MBC, indicating a strong effect of natural ABA. The MBC test can be used to evaluate formulation problems when there are suspicions that the active ingredient is being “bound up” by other ingredients. According to the MBC/MIC ratio, we assessed antibacterial activity. The MBC/MIC ratio is used to evaluate antibacterial activity. If the MBC/MIC ratio is ≤4, the effect is judged to be bactericidal, and if the MBC/MIC ratio is > 4, the effect is considered to be bacteriostatic.

In the study of Pavlović et al. (2017) [28], five various WGEs demonstrated certain ABA against all tested enteropathogenic bacteria, with MIC values ranging from 1.56 to 25.00 mg/mL and MBC values from 3.13 to 50.00 mg/mL. The most noticeable effect in the case of *S. enteritidis* was achieved for 96% ethanol WGE (MIC/MBC = 1.56/3.13 mg/mL). These results are in accordance with the findings [58] that the WG leaf extract inhibits the growth of *E. coli, S. aureus* and *Salmonella enterica*. A water extract (at pH 7.0, adjusted with 0.1 mol/L K_2_HPO_4_) from WG leaves exhibited ABA in vitro on *Listeria monocytogenes, S. aureus, E. coli*, and *Salmonella enterica subsp. enterica* with a larger diameter of inhibition zones in the case of Gram (+) bacteria [58]. WG methanol extracts showed a more powerful ABA than the watery one (at the concentration range 0.06–35.5 mg/mL and 0.16–83.7, respectively). It inhibited the growth of *Staphylococcus aureus*, *Bacillus subtilis*, *Escherichia coli, Proteus mirabilis*, *and Salmonella enteritidis* [59]. Allicin is the most important and the most active substance detected in the fresh WG leaf extract [60]. The mechanism of the ABA of sulfur compounds is complex and insufficiently explained. It is generally recognized that the ABA of sulfur compounds depends on their hydrophilic or lipophilic character [60]. Herbal medicines are the cheapest way of treatment for various diseases, as they can be easily prepared and bought over the counter, outside pharmacies [61]. It is quite true that the integration of herbal medicines into the primary health care system of developing countries is expanding; for this very reason, the issue of the safety of natural sources of bioactive substances must not be neglected [61]. The authors revealed that filtration is a simple, cheap, and successful method of removing undesirable microorganisms from WGE. This contributes to the initiative to connect food business operators and national food safety authorities to synergistically lay the ground for creating products that are safe for consumers [62]. A group of authors [52] tested the ABA of acetone, chloroform, ethyl acetate, n-butanol, and water extracts of fresh flowers and leaves of *A. ursinum*. None of the extracts showed any inhibition of *E. coli*. Acetone and chloroform extracts from both parts of the WG showed good inhibition of *S. aureus*. Some authors state that the average MIC value was 35 mg/mL, except for the *S. aureus* ATTC 25923 strain (MIC = 17.7 mg/mL) [59], while other authors reported that the inhibition zones were greater in the case of Gram (+) bacteria [27].

Herbal extracts may be used as possible sources to obtain new and effective medicines to treat food-borne diseases, or an excellent alternative to combat the further spread of multi-drug-resistant microorganisms [63]. The use of phenol ingredients as antimicrobials has dual-function potency: preservation of food and health benefits [63]. The results emphasized the importance of phenol compounds in the ABA of herbal extracts and also indicated that the phenol compounds significantly contributed to their ABA [64]. The ABA of the *Allium* species is predominantly associated with the alk(en)yl alka/ene thiosulfinates and some products of their transformation and polyphenolic substances [54,65,66]. In our FSWGEs, the content of allicin was determined by HPLC (11.375 mg/mL), in contrast to extracts obtained by subcritical water extraction, where allicin was not isolated (it is assumed that this is due to the thermolability of the sulfur compounds and to the fact that high temperatures are used during extraction and high allicin instability). The ABA of phenols is not fully clarified, and it is known that there are several sites of their potential action at the cellular level, such as causing irreversible changes in membrane proteins of *E. coli*, *P. aeruginosa*, *S. aureus,* and *L. monocytogenes* that manifest as a strong antibacterial effect of gallic acid [67]. The ABA mainly depends on the position of the hydroxyl and carboxyl groups, and the double bonds present in the phenol ring dictate the strength of their ABA [68]. In a larger number of studies, contradictory results were obtained for the ABA of WGE against the tested Gram (+) and Gram (–) bacteria, which was interpreted by isolating different active compounds using different solvents during extraction, extraction methods, plant-origin, and plant parts [31,52,69,70]. We took advantage of the well-known fact that WG contains about seven times more active sulfur compounds than garlic [71] and replaced garlic as a spice for BUs (or some other meat product) with WGE, which shows powerful AOX and ABA important for the prolonged shelf life and safety of the product, as well as the listed benefits for human health. To the best of our knowledge, this is the first comprehensive examination of the impact of WG leaf extract on the techno-functional properties of BUs as a food model system. The idea behind our concept is based on the proven positive effects of WGE bioactive substances on human health, confirmed in modern research of the traditional use of WG. Flavonoids inhibit platelet aggregation in humans and possess AOX activity [72,73]. Kaempferol and its glycosides have an anti-inflammatory, antimicrobial, anticancer, cardioprotective, neuroprotective, anti-diabetic, anti-allergic, and anti-asthmatic effect, and they also contribute to the prevention of hypercholesterolemia and high blood pressure [74,75]. The sulfur compounds have shown anticancer, antimicrobial, cardioprotective, antihypertensive, and anti-aggregation effects and ACE inhibition [25,28,48,58,60,76,77,78,79,80,81].

### 3.3. Total Phenolics and Antioxidant Capacity in Raw Burgers

Results presented in Table 5 revealed that varying the level of freshly squeezed wild garlic extract (FSWGE) resulted in no statistically significant differences in the content of polyphenolics in freshly prepared (day 0) raw burgers (BUs) and in the same BUs after 10 days of cold storage (day 10). On the other hand, the concentration of FSWGEs in BUs affected the level of polyphenols on days 5 and 90 of freeze storage. Namely, Bus with the highest amount of FSWGEs added (PS-III) had the highest content of polyphenolics, while the lowest content of polyphenols was found in PS-I burgers after five days of cold storage. In contrast, after 90 days of freezing, the lowest content of polyphenolics was detected in PS-III Bus. As for the antioxidant (AOX) capacity, certain changes among BU categories were noticed. BU of PS-III had the highest level of AOX capacity (slower oxidation rates) in all three testing dates (days 0, 5, and 10). WG extract exerted significant antioxidant effects [82]. The addition of FSWGEs did not influence the total level of polyphenols after 90 days of freeze-storage. Burgers of PS-III indicate a stronger AOX activity of FSWGEs in relation to garlic, which is commonly used to season BUs and was added as a spice exclusively to control group (CON) samples.

Determination of the phenolic profile and diallyl thiosulfonate (allicin) in herb extract by HPLC allows a precise definition of the application dose range, according to the literature data. The maximum recommended dose of WGE for use in foodstuffs is 5% (*w/w*). For a food product, a minimum dose is 1 to 5 ppm, and a maximum dose is 5% (*w/w*) (Patent Application Publication Pub. No.: US 2007/0160725 A1, 2007, United States). Dissolved WGE applied in the effective concentration of 8.79 mL WGE/kg in samples of PS-III Bus revealed stronger AOX action than BUs modified with lower concentrations of WGEs (4.40 mL and 1.32 mL WGE/kg of BU in PS-II and PS-I, respectively). The explanation for the significantly higher level of AOX activity in BU samples of those production series modified by the addition of different concentrations of WGE can be found in the fact that the AOX effect may have organosulfur compounds abundant in freshly squeezed WGE, shown in Table 1 and Table 2. The results are even more significant due to the fact that fat from frozen meat and solid fat (used in the BU preparation within this study) easily oxidized, and AOX effectiveness was reduced in products with higher fat content [83]. Duration of cold storage showed no influence on polyphenol content within all four BU categories, while freeze storage for 90 days resulted in a decrease in polyphenols within CON and PS-III BU categories. Prolonging cold storage resulted in a decrease in AOX capacity in all categories except PS-II, whilst 90 days of freeze storage resulted in an increase in AOX potency in all four BU categories. Freezing BU samples to −20 °C resulted in the creation of ice crystals, which might damage the meat tissue.

Thus, certain phenolic compounds might be released from the tissue and could consequently increase the AOX capacity. On the other hand, decreasing storage temperature led to a decrease in osmotic pressure, which is an onward constant during the entire storage period of 90 days. Subsequent warming of frozen BU samples to room temperature in order to perform the extractions caused a drying-like effect, in which a sudden increase of the osmotic pressure initiated the migration of phenolic compounds conjointly with water from the tissue towards the sample surface. Such water migration, accompanied by solutes from the inner parts to the surface, is a well-known phenomenon during fruit and vegetable drying [84]. Most likely, these two phenomena mutually caused an increase in AOX capacity. Antonini et al., 2020 [20] determined the level of polyphenolic in beef BUs with the addition of chia seeds and goji puree. The results revealed 20.9 mg/100 g in the control sample and up to 34 mg/100 g in BUs with supplements. Nonetheless, the addition of chia seeds or goji puree has certainly led to an increase in polyphenol content and AOX capacity (ranging from 0.132 mmol TE/100 g in the control sample up to 0.236 mmol TE/100 g in BU with goji and chia addition). Such an increase is ascribed to the synergistic effect of AOX molecules of goji and chia seeds with hydrophilic and lipophilic AOX of beef meat (carnosine, anserine, L-carnitine, glutathione, taurine, creatine, etc.). On the other hand, our results showed that adding FSWGE in raw beef/pork BUs did not necessarily increase the total content of phenolics or the AOX capacity. However, the levels are comparable (40 to 81 mg/100 g and 0.283 to 0.563 mmol TE/100 g for polyphenolics and antioxidant activity, respectively) and slightly increased relative to those results of the authors [20]. Higher AOX efficiency of phenolic-rich crude extracts compared to pure phenolic compounds has been documented in the literature [85,86].

### 3.4. Technological Properties and Proximate Composition of Burgers

The addition of FSWGE to burger (BU) formulation reduces weight loss (WL) (Table 6). However, no effect of increasing the content of FSWGE on WL was observed. During cold and freeze storage, a slight decrease in WL values was observed; however, without significant differences, leading to the same relations within treatments on day 10 (end of cold storage) and day 90 (end of freeze storage) as at the beginning of storage (day 1)—namely, significantly lower WL values were obtained in BUs with FSWGE compared to CON. Diameter reduction (dR) together with WL can indicate BU deformation during grilling [87]. No significant influence of FSWGE amount and storage time during both cold and freeze storage was observed, though, after freeze storage, lower values of dR were measured after grilling before and after storage, and within all treatments. Though WL was significantly lower in all BUs with FSWGE, this did not alter the proximate composition of grilled BUs (Table 6). Other research also reported that the addition of plant extracts on BU/patty-type meat products did not alter their proximate composition and product deformation during grilling [88,89].

pH values (Table 6) of raw BUs were higher in burgers with FSWGE, and though significant differences were observed between CON and PS-II, they were within the range reported for beef and pork BUs [15,90]. During cold storage, a significant increase in pH value in CON was observed (day 5), followed by a significant decrease at the end of storage, reaching the value that did not differ from day 1. Within BUs with FSWGE, the opposite pattern was observed. However, on day 10, similar relations between treatments were observed as on day 1. A group of authors [91] observed a similar pattern when adding tea and natural grape extracts to pork patties throughout storage. The pH values of all treatments after freeze storage were significantly higher, though without differences within them. After grilling, pH values (in both cooled and frozen BUs) were higher in all treatments, which was expected [92,93], without significant differences between treatments (except on day 5).

### 3.5. Sensory Analysis

The addition of FSWGE in different amounts did not reduce the sensory acceptance of modified BUs (Figure 1). On the contrary, except in terms of odor, where PS-I and PS-III had slightly lower grades (7.03 and 7.15, respectively) compared to control (7.18), all BUs with FSWGE received higher scores. Moreover, regarding color, assessors gave PS-III significantly higher grades than control, while PS-II received significantly higher grades than control regarding texture and overall acceptance. There were no significant differences between CON and modified BUs in terms of odor and taste. No significant differences were observed within BUs with FSWGE regarding all observed sensory properties. Research by other authors shows that the addition of red pitaya extract to pork patties (with the total replacement of animal fat) did not alter the sensory properties and, similarly to our findings, they reported that color-wise, consumers gave significantly higher grades to the treatment with the highest content of plant extract than control [89]. Additionally, research shows that the addition of pitanga leaf extracts did not change the acceptability of cooked lamb BUs, where fat was replaced entirely with chia oil [88]. The results of this research indicate that, in addition to improving the oxidative and microbiological stability of BUs, FSWGEs can be successfully used as a substitute for garlic, as a spice and in commercial spice mixtures.

## 4. Conclusions

The results of this study have demonstrated the great potential of wild garlic extract in preserving burgers during cold storage. The determined optimal concentration of dissolved *Allium ursinum* L. extract (10% w/v, applied in an effective concentration of 12.5 g/kg of minced meat) revealed the strongest activity by using different contemporary methods for testing antioxidative effects. Freshly squeezed wild garlic extract (FSWGE) exhibited the strongest antibacterial activity against *E. coli*, *S. aureus*, *S. enteritidis*, *B. subtilis*, and *L. monocytogenes*. In contrast, *S. marcescens*, *L. monocytogenes*, *E. coli*, and *S. aureus* exhibited the highest susceptibility to the evaporated FSWGE. Weight loss after grilling was reduced in burgers with FSWGE. However, this did not alter the proximate composition of grilled burgers. The addition of FSWGE did not reduce the sensory quality of burgers—on the contrary, all modified burgers received higher scores in terms of color, taste, texture and overall acceptance.

It is necessary to improve the research on the use of wild garlic extract and other herb extracts as preservatives in future and to promote new approaches, such as the use of a low-dose synergistic antimicrobial combination of plant extracts (phytocomplexes).

## Figures and Tables

**Table 1:** Detected and quantified compounds in FSWGE by LC-MS/MS.

Compound [Molecular Formula]	RT ^1^ (min)	Concentration (mg/mL)
Allicin [C_6_H_10_OS_2_]	6.21	11.375
Ferulic acid [C_10_H_10_O_4_]	5.53	4.259
p-Coumaric acid [C_9_H_8_O_3_]	4.86	1.453
Sinapic acid [C_11_H_12_O_5_]	5.27	1.175

^1^ RT—Retention times; FSWGE—freshly squeezed wild garlic extracts.

**Table 2:** Volatile compounds in FSWGE.

	Compound [Molecular Formula]	RT ^1^ (min)	Area (%) ^2^
1.	Methyl 2-propenyl trisulfide [C_4_H_8_S_3_]	13.233	21.63
2.	Diallyl disulfide [C_6_H_10_S_2_]	10.757	13.30
3.	Diallyl trisulfide [C_6_H_10_S_3_]	20.305	12.87
4.	Allyl methyl disulfide [C_4_H_8_S_2_]	5.428	8.43
5.	Dimethyl trisulfide [C_2_H_6_S_3_]	6.881	4.66
6.	(3-Chlorophenyl)acetylene [C_8_H_5_Cl]	15.602	3.80
7.	2-Ethylbenzenesulfonamide [C_8_H_11_NO_2_S]	6.482	3.29
8.	Ethyl vinyl disulfide [C_4_H_8_S_2_]	23.710	3.16
9.	Allyl-1-propenyl trisulfide [C_6_H_10_S_3_]	21.374	2.58
10.	Isobutyl isothiocyanate [C_5_H_9_NS]	20.754	2.00
11.	Diallyl tetrasulfide [C_6_H_10_S_4_]	30.399	1.92
12.	1-Methyl-3-(methylamino)-4-pyrazolecarboxamide [C_6_H_10_N_4_O]	13.604	1.56
13.	Methyl 1-propenyl disulfide [C_4_H_8_S_2_]	5.982	1.51
14.	3-Vinyl-4H-1,2-dithiin [C_6_H_8_S_2_]	15.120	1.08
15.	Dimethyl disulfide [C_2_H_6_S_2_]	2.700	0.92
16.	2-Vinyl-4H-1,3-dithiine [C_6_H_8_S_2_]	16.223	0.88
17.	Dihydro-2(3H)-thiophenthione [C_4_H_6_S_2_]	11.387	0.78
18.	3-Sulfanyl-2-(sulfanylmethyl)propanoic acid [C_4_H_8_O_2_S_2_]	14.004	0.63
19.	Methyl 2-(propylthio)acetate [C_6_H_12_O_2_S]	12.104	0.36
20.	Hexathiepane [CH_2_S_6_]	36.888	0.34
21.	2-Chloro-6-(methoxymethyl)toluene [C_9_H_11_ClO]	34.193	0.34
22.	1,2-Benzenediamine, N,N’-disulfinyl [C_6_H_4_N_2_O_2_S_2_]	34.858	0.32
23.	Dimethyl tetrasulfide [C_2_H_6_S_4_]	19.837	0.32
24.	2,2,4,6,6-Pentamethylheptane [C_12_H_26_]	7.420	0.19
25.	Triphenylboroxin[C_18_H_15_B_3_O_3_]	45.592	0.15
26.	3-Methylbutanal [C_5_H_10_O]	50.582	0.14
27.	Propachlor [C_11_H_14_ClNO]	21.959	0.14
28.	2,4-Dimethylthiophene [C_6_H_8_S]	5.130	0.13
29.	Dimethyl pentasulfide [C_2_H_6_S_5_]	33.535	0.12
30.	Diallyl sulfide [C_6_H_10_S]	4.173	0.11
31.	Pyrimidine-2,4(1H,3H)-dione [C_4_H_4_N_4_O_3_]	50.932	0.09
32.	2-Hexylthiirane [C_8_H_16_S]	10.367	0.08
33.	Methyl octadeca-9,12,15-trienoate [C_19_H_32_O_2_]	52.669	0.08
34.	1,4,7-Trithionane 1,1-dioxide [C_6_H_12_O_2_S_3_]	29.714	0.07
35	1,4-Dithiepane-2-butanal, 3-oxo-[C_9_H_14_O_2_S_2_]	32.972	0.05
36.	2,2,4,4-Tetramethyloctane [C_12_H_26_]	8.683	0.02
37.	Methanethiol, N-cyclopropylamidino-, hydrogen thiosulfate [C_5_H_10_N_2_O_3_S_2_]	41.571	0.02
38.	Hexanal [C_6_H_12_O]	3.255	0.01
Total Identified			88.08

^1^ RT—Retention times. ^2^ The contents (%) of the individual components were calculated based on the peak area (FID response). FSWGE—freshly squeezed Wild Garlic extracts.

**Table 3:** Antibacterial activity of FSWGE (%).

Tested Bacteria/Strain	MIC (%)	MBC (%)
Enterococcus faecium ATCC 6057	50	>60
2. Listeria monocytogenes ATCC 13932	30	>50
3. Staphylococcus aureus ATCC 25923	30	40
4. Salmonella enteritidis ATCC 13076	30	40
5. Escherichia coli WDCM 0013	20	30
6. Bacillus subtilis ATCC 6633	30	40

FSWGE—Freshly squeezed Wild Garlic extracts.

**Table 4:** Antibacterial activities of EWGE (mg/mL).

Tested Bacteria/Strain	MIC (mg/mL)	MBC (mg/mL)
Enterococcus faecium ATCC 6057	100	>100
2. Listeria monocytogenes ATCC 13932	90	90
3. Staphylococcus aureus ATCC 25923	90	>100
4. Salmonella enteritidis ATCC 13076	100	>100
5. Escherichia coli WDCM 0013	90	100
6. Serratia marcescens ATCC 43862	50	60

EWGE—evaporated freshly squeezed Wild Garlic extracts (FSWGE); MIC—minimum inhibitory concentrations; MBC—minimum bactericidal concentration.

**Table 5:** Effect of cold and freeze storage on total phenolics and AOX capacity of control and burgers fortified with different concentrations of FSWGA.

	Production Series	CON	PS-I	PS-II	PS-III
	**Storage** **Duration**	**Cold Storage**			
Total phenolics (mg/100 g of BU)	Day 0	81.39 ± 11.77	69.30 ± 9.69	73.41 ± 3.61	71.55 ± 5.63
	Day 5	76.16 ^ab^ ± 5.33	60.47 ^a^ ± 9.40	77.17 ^ab^ ± 5.96	81.32 ^b^ ± 7.63
	Day 10	77.78 ± 5.87	72.93 ± 3.82	72.33 ± 5.94	63.65 ± 6.67
AOX capacity (ABTS, mmol TE/100 g of BU)	Day 0	0.420 ^bcB^ ± 0.006	0.392 ^abC^ ± 0.007	0.353 ^a^ ± 0.006	0.442 ^cB^ ± 0.037
	Day 5	0.291 ^aA^ ± 0.002	0.339 ^bB^ ± 0.008	0.320 ^b^ ± 0.002	0.341 ^bA^ ± 0.036
	Day 10	0.321 ^abA^ ± 0.024	0.283 ^aA^ ± 0.004	0.319 ^ab^ ± 0.009	0.347 ^bA^ ± 0.007
		**Freeze Storage**			
Total phenolics (mg/100 g of BU)	Day 0	81.39 ^B^ ± 11.77	69.30 ± 9.69	73.41 ± 3.61	71.55 ^B^ ± 5.63
	Day 90	56.65 ^bA^ ± 2.50	65.91 ^b^ ± 6.60	66.17 ^b^ ± 4.03	40.48 ^aA^ ± 2.05
AOX capacity (ABTS, mmol TE/100 g of BU)	Day 0	0.420 ^bcA^ ± 0.006	0.392 ^abA^ ± 0.007	0.353 ^aA^ ± 0.006	0.442 ^cA^ ± 0.037
	Day 90	0.553 ^B^ ± 0.015	0.563 ^B^ ± 0.011	0.545 ^B^ ± 0.015	0.540 ^B^ ± 0.011

CON—without FSWGE; PS-I with 1.32 mL of FSWGE/kg of BU; PS-II with 4.40 mL of FSWGE/kg of BU; PS-III with 8.79 mL of FSWGE/kg of BU; FSWGE—freshly squeezed wild garlic ex00.tracts; BU—burger. ^a–c^ Values (mean ± SD) in the same row with different superscripts are significantly different (*p* < 0.05). ^A–C^ Uppercase letters are used for comparing the samples considering the effect of storage. Values in the same column for the same property, with different superscripts, are significantly different (*p* < 0.05).

**Table 6:** Technological properties and proximate composition * of burgers.

		CON	PS-I	PS-II	PS-III
Cold Storage					
WL (%)	Day 1	17.01 ^bB^ ± 0.50	14.26 ^a^ ± 1.23	13.06 ^a^ ± 0.53	13.53 ^a^ ± 0.19
	Day 5	14.26 ^A^ ± 1.51	13.71 ± 0.98	12.69 ± 0.70	12.77 ± 0.67
	Day 10	16.04 ^bAB^ ± 1.16	13.14 ^a^ ± 0.81	13.20 ^a^ ± 0.87	12.98 ^a^ ± 1.01
dR (%)	Day 1	19.21 ± 1.65	20.04 ± 1.94	17.28 ± 0.81	18.91 ± 1.36
	Day 5	18.68 ± 1.44	19.62 ± 1.52	20.38 ± 2.42	20.68 ± 1.67
	Day 10	16.70 ± 1.16	21.10 ± 1.90	20.18 ± 1.54	17.76 ± 3.30
pH raw	Day 1	6.47 ^aAB^ ± 0.03	6.55 ^ab^ ± 0.03	6.59 ^bB^ ± 0.04	6.56 ^abB^ ± 0.04
	Day 5	6.57 ^bB^ ± 0.04	6.49 ^b^ ± 0.09	6.37 ^aA^ ± 0.06	6.30 ^aA^ ± 0.10
	Day 10	6.38 ^aA^ ± 0.07	6.51 ^b^ ± 0.09	6.46 ^abA^ ± 0.07	6.46 ^abB^ ± 0.11
pH grilled	Day 1	6.70 ^B^ ± 0.05	6.70 ^B^ ± 0.02	6.70 ^B^ ± 0.04	6.69 ^B^ ± 0.03
	Day 5	6.74 ^bB^ ± 0.03	6.59 ^aA^ ± 0.06	6.55 ^aA^ ± 0.04	6.53 ^aA^ ± 0.05
	Day 10	6.56 ^A^ ± 0.07	6.60 ^A^ ± 0.07	6.57 ^A^ ± 0.06	6.61 ^AB^ ± 0.06
**Freeze Storage**					
WL (%)	Day 1	17.01 ^b^ ± 0.50	14.26 ^a^ ± 1.23	13.06 ^a^ ± 0.53	13.53 ^a^ ± 0.19
	Day 90	15.13 ^b^ ± 1.26	12.73 ^a^ ± 0.46	12.11 ^a^ ± 1.01	12.03 ^a^ ± 0.78
dR (%)	Day 1	19.21 ± 1.65	20.04 ± 1.94	17.28 ± 0.81	18.91 ± 1.36
	Day 90	16.48 ± 1.56	16.25 ± 1.80	15.36 ± 0.95	14.05 ± 1.12
pH raw	Day 1	6.47 ^aA^ ± 0.03	6.55 ^abA^ ± 0.03	6.59 ^bA^ ± 0.04	6.56 ^abA^ ± 0.04
	Day 90	6.76 ^B^ ± 0.05	6.74 ^B^ ± 0.07	6.74 ^B^ ± 0.06	6.75 ^B^ ± 0.06
pH grilled	Day 1	6.70 ^A^ ± 0.05	6.70 ^A^ ± 0.02	6.70 ^A^ ± 0.04	6.69 ^A^ ± 0.03
	Day 90	6.81 ^B^ ± 0.05	6.82 ^B^ ± 0.04	6.84 ^B^ ± 0.01	6.84 ^B^ ± 0.04
**Proximate Composition**					
moisture	Day 1	56.72 ± 0.58	56.90 ± 0.93	57.20 ± 0.56	57.15 ± 0.48
protein		20.75 ± 1.92	20.87 ± 0.65	19.89 ± 0.48	20.90 ± 0.27
fat		15.97 ± 0.87	16.07 ± 0.83	17.37 ± 1.02	17.30 ± 0.82

* Grilled, day 1; CON—without FSWGE; PS-I with 1.32 mL of FSWGE/kg of BU; PS-II with 4.40 mL of FSWGE/kg of BU; PS-III with 8.79 mL of FSWGE/kg of BU; FSWGE—freshly squeezed Wild Garlic extracts; BU—burger; WL—weight loose; dR—diameter reduction. ^a,b^ Values (mean±SD) in the same row with different superscripts are significantly different (*p* < 0.05). ^A,B^ Uppercase letters are used for comparing the samples considering the effect of storage. Values in the same column for the same property, with different superscripts, are significantly different (*p* < 0.05).

## Data Availability

Data sharing is not applicable to this article.

## References

[1] Bhaskar R., Ola M. (2012). Junk food: Impact on health. J. Drug Deliv. Sci. Technol..

[2] Bhushan C., Taneja S., Khurana A. Centre for Science and Environment, New Delhi, Burden of Packaged Food on Schoolchildren: Based on the CSE Survey ‘Know Your Diet’. https://www.jstor.org/stable/resrep38038.

[3] Singh S.A., Dhanasekaran D., Ganamurali N.L.P., Sabarathinam S. (2021). Junk food-induced obesity—A growing threat to youngsters during the pandemic. Obes. Med..

[4] Jiang J., Xiong Y.L. (2016). Natural antioxidants as food and feed additives to promote health benefits and quality of meat products: A review. Meat Sci..

[5] Fuhrman J. (2018). The hidden dangers of fast and processed food. Am. J. Lifestyle Med..

[6] WHO The Top 10 Causes of Death. https://www.who.int/news-room/fact-sheets/detail/the-top-10-causes-of-death.

[7] Patibandla G., Yamani L. (2021). Fast food and soft drink consumption pattern in medical students and its association with overweight and obesity. Glob. J. Med. Stud..

[8] da Silva R.P., Rocha-Santos T.A., Duarte A.C. (2016). Supercritical fluid extraction of bioactive compounds. Trends Analyt. Chem..

[9] Łuczaj Ł., Pieroni A., Sánchez-Mata M., Tardío J. (2016). Nutritional ethnobotany in Europe: From emergency foods to healthy folk cuisines and contemporary foraging trends. Mediterranean Wild Edible Plants.

[10] Aschemann-Witzel J., Varela P., Peschel A.O. (2019). Consumers’ categorization of food ingredients: Do consumers perceive them as ‘clean label’ producers expect? An exploration with projective mapping. Food Qual. Prefer..

[11] Bis-Souza C.V., Barba F.J., Lorenzo J.M., Penna A.L.B., Barretto A.C.S. (2019). New strategies for the development of innovative fermented meat products: A review regarding the incorporation of probiotics and dietary fibers. Food Rev. Int..

[12] Granato D., Barba F.J., Kovačević D.B., Lorenzo J.M., Cruz A.G., Putnik P. (2020). Functional foods: Product development, technological trends, efficacy testing, and safety. Annu. Rev. Food Sci. Technol..

[13] Lee S.Y., Lee D.Y., Kim O.Y., Kang H.J., Kim H.S., Hur S.J. (2020). Overview of studies on the use of natural antioxidative materials in meat products. Food Sci. Anim. Resour..

[14] Yong H.I., Kim T.K., Choi H.D., Jung S., Choi Y.S. (2020). Technological strategy of clean label meat products. Food Life.

[15] Heck R.T., Vendruscolo R.G., de Araújo Etchepare M., Cichoski A.J., de Menezes C.R., Barin J.S., Lorenzo J.M., Wagner R., Campagnol P.C.B. (2017). Is it possible to produce a low-fat burger with a healthy n-6/n-3 PUFA ratio without affecting the technological and sensory properties?. Meat Sci..

[16] Gyawali R., Ibrahim S.A. (2014). Natural products as antimicrobial agents. Food Control.

[17] Inetianbor J., Yakubu J., Ezeonu S. (2016). Effects of food additives and preservatives on man: A review. AJST.

[18] Lee N.-K., Paik H.-D. (2016). Status, antimicrobial mechanism, and regulation of natural preservatives in livestock food fystems. Food Sci. Anim. Resour..

[19] Sharif Z.I.M., Mustapha F.A., Jai J., Yusof N., Zaki N.A.M. (2017). Review on methods for preservation and natural preservatives for extending the food longevity. Chem. Eng. Res. Bull..

[20] Antonini E., Torri L., Piochi M., Cabrino G., Melia M.A., Bellis R.D. (2020). Nutritional, antioxidant and sensory properties of functional beef burgers formulated with chia seeds and goji puree, before and after in vitro digestion. Meat Sci..

[21] Vargas-Ramella M., Munekata P.E.S., Pateiro M., Franco D., Campagnol P.C.B., Tomasevic I., Domínguez R., Lorenzo J.M. (2020). Physicochemical composition and nutritional properties of deer burger enhanced with healthier oils. Foods.

[22] Ziegler V., Ugalde M.L., Veeck I.A., Barbosa F.F. (2020). Nutritional enrichment of beef burgers by adding components of non-conventional food plants. Braz. J. Food Technol..

[23] Voća S., Šic Žlabur J., Fabek Uher S., Peša M., Opačić N., Radman S. (2022). Neglected potential of wild garlic (*Allium ursinum* L.)—Specialized metabolites content and antioxidant capacity of wild populations in relation to location and plant phenophase. Horticulturae.

[24] Sharma S.K., Singh L., Singh S. (2013). A review on medicinal plants having antioxidant potential. Indian J. Res. Pharm. Biotechnol..

[25] Radulović N.S., Miltojević A.B., Stojković M.B., Blagojević P.D. (2015). New volatile sulfur-containing compounds from wild garlic (*Allium ursinum* L., Liliaceae). Food Res. Int..

[26] Djurdjevic L., Dinic A., Pavlovic P., Mitrovic M., Karadzic B., Tesevic V. (2004). Allelopathic potential of *Allium ursinum* L. Biochem. Syst. Ecol..

[27] Sobolewska D., Podolak I., Makowska-Was J. (2015). *Allium ursinum*: Botanical, phytochemical and pharmacological overview. Phytochem. Rev..

[28] Pavlović D.R., Veljković M., Stojanović N.M., Gočmanac-Ignjatović M., Mihailov-Krstev T., Branković S., Radenković M. (2017). Influence of different wild-garlic (*Allium ursinum*) extracts on the gastrointestinal system: Spasmolytic, antimicrobial and antioxidant properties. J. Pharm. Pharmacol. J..

[29] Shahidi F., Hossain A. (2018). Bioactives in spices, and spice oleoresins: Phytochemicals and their beneficial effects in food preservation and health promotion. J. Food Bioact..

[30] Stanisavljević N., Soković Bajić S., Jovanović Ž., Matić I., Tolinački M., Popović D., Samardžić J. (2020). Antioxidant and antiproliferative activity of *Allium ursinum* and their associated microbiota during simulated in vitro digestion in the presence of food matrix. Front. Microbiol..

[31] Rankovic M., Krivokapic M., Bradic J., Petkovic A., Zivkovic V., Sretenovic J., Tomovic M. (2021). New insight into the cardioprotective effects of *Allium ursinum* L. extract against myocardial ischemia-reperfusion injury. Front. Physiol..

[32] Coulston A.M., Rock C.L., Monsen E.R. (2001). Nutrition in the Prevention and Treatment of Disease.

[33] Wu H., Dushenkov S., Ho C.-T., Sang S. (2009). Novel acetylated flavonoid glycosides from the leaves of *Allium ursinum*. Food Chem..

[34] Gîtin L., Dinică R., Parnavel R. (2012). The influence of extraction method on the apparent content of bioactive compounds in Romanian Allium spp. leaves. Not. Bot. Horti Agrobot. Cluj-Napoca.

[35] Oszmiański J., Kolniak-Ostek J., Wojdyło A. (2013). Characterization and content of flavonol derivatives of *Allium ursinum* L. plant. J. Agric. Food Chem..

[36] Piatkowska E., Kopeć A., Leszczynska T. (2015). Basic chemical composition, content of micro and macro elements and antioxidant activity of different varieties of garlic’s leaves polish origin. ZYWNOSC—Nauka Technol. Jakosc.

[37] Lachowicz S., Oszmiański J., Wiśniewski R. (2018). Determination of triterpenoids, carotenoids, chlorophylls, and antioxidant capacity in *Allium ursinum L*. at different times of harvesting and anatomical parts. Eur. Food Res. Technol..

[38] Śmiecińska K., Gugołek A., Kowalska D. (2022). Effects of garlic (*Allium sativum* L.) and ramsons (*Allium ursinum* L.) on lipid oxidation and the microbiological quality, physicochemical properties and sensory attributes of rabbit meat burgers. Animals.

[39] Spencer E.H., Frank E., McIntosh N.F. (2005). Potential effects of the next 100 billion hamburgers sold by McDonald’s. Am. J. Prev. Med..

[40] Thiers B. (2021). Index Herbariorum: A Global Directory of Public Herbaria and Associated Staff. New York Botanical Garden’s Virtual Herbarium. http://sweetgum.nybg.org/ih.

[41] Singleton V., Orthofer R., Lamuela-Raventos R.M. (1999). Analysis of total phenols and other oxidation substrates and antioxidants by means of Folin-Ciocalteu reagent. Methods Enzymol..

[42] Liu M., Li X.Q., Weber C., Lee C.Y., Brown J., Liu R.H. (2002). Antioxidant and antiproliferative activities of raspberries. J. Agric. Food Chem..

[43] Re R., Pellegrini N., Proteggente A., Pannala A., Yang M., Rice-Evans C. (1999). Antioxidant activity applying an improved ABTS radical cation decolorization assay. Free Radic. Biol. Med..

[44] (1997). Meat and Meat Products—Determination of Moisture Content.

[45] (1978). Meat and Meat Products. Determination of Nitrogen Content.

[46] (1973). Meat and Meat Products. Determination of Total Fat Content.

[47] Poojary M.M., Putnik P., Bursać Kovačević D., Barba F.J., Lorenzo J.M., Dias D.A., Shpigelman A. (2017). Stability and extraction of bioactive sulfur compounds from *Allium* genus processed by traditional and innovative technologies. J. Food Compos. Anal..

[48] Putnik P., Gabrić D., Roohinejad S., Barbad F.J., Granato D., Mallikarjunanb K., Bursać Kovačević D. (2019). An overview of organosulfur compounds from *Allium* spp.: From processing and preservation to evaluation of their bioavailability, antimicrobial, and anti-inflammatory properties. Food Chem..

[49] Vlase L., Parvu M., Parvu E.A., Toiu A. (2013). Phytochemical analysis of *Allium fistulosum* L. and *A. ursinum* L. Dig. J. Nanomater. Biost..

[50] Pop R.M., Bocsan I.C., Buzoianu A.D., Chedea V.S., Socaci S.A., Pecoraro M., Popolo A. (2020). Evaluation of the antioxidant activity of *Nigella sativa* L. and *Allium ursinum* extracts in a cellular model of doxorubicin-induced cardiotoxicity. Molecules.

[51] Petropoulos S.A., Di Gioia F., Polyzos N., Tzortzakis N. (2020). Natural antioxidants, health effects and bioactive properties of wild allium species. Curr. Pharm. Des..

[52] Ivanova A., Mikhova B., Najdenski H., Tsvetkova I., Kostova I. (2009). Chemical composition and antimicrobial activity of wild garlic *Allium ursinum* of Bulgarian origin. Nat. Prod. Commun..

[53] Gođevac D., Vujisić L., Mojović M., Ignjatović A., Spasojević I., Vajs V. (2008). Evaluation of antioxidant capacity of *Allium ursinum* L. volatile oil and its effect on membrane fluidity. Food Chem..

[54] Vidović S., Tomšik A., Vladić J., Jokić S. (2021). Supercritical carbon dioxide extraction of *Allium ursinum*: Impact of temperature and pressure on the extracts chemical profile. Chem. Biodivers..

[55] Schmitt B., Schulz H., Storsberg J., Keusgen M. (2005). Chemical characterization of *Allium ursinum* L. depending on harvesting time. J. Agric. Food Chem..

[56] Błażewicz-Woźniak M., Michowska A. (2011). The growth, flowering and chemical composition of leaves of three ecotypes of *Allium ursinum* L. Acta Agrobot..

[57] Kim S.-J., Cho A.R., Han J. (2013). Antioxidant and antimicrobial activities of leafy green vegetable extracts and their applications to meat product preservation. Food Contr..

[58] Sapunjieva T., Alexieva I., Mihaylova D., Popova A. (2012). Antimicrobial and antioxidant activity of extracts of *Allium ursinum* L. J. BioSci. Biotechnol..

[59] Synowiec A., Gniewosz M., Zieja I., Baczek K., Przybyl J. (2010). The comparison of antimicrobial properties of ramson (*Allium ursinum*) extracts. Zesz. Probl. Postep. Nauk Roln..

[60] Bagiu R.V., Vlaicu B., Butnariu M. (2012). Chemical composition and in vitro antifungal activity screening of the *Allium ursinum* L. (Liliaceae). Int. J. Mol. Sci..

[61] de Sousa Lima C.M., Fujishima M.A.T., de Paula Lima B., Mastroianni P.C., de Sousa F.F.O., da Silva J.O. (2020). Microbial contamination in herbal medicines: A serious health hazard to elderly consumers. BMC Complement. Med. Ther..

[62] Kurćubić V.S., Stajić S.B., Dmitrić M.P., Miletić N.M. (2022). Food safety assessment of burger patties with added herbal plant material. Fleischwirtschaft.

[63] Fullerton M., Khatiwada J., Johnson J.U., Davis S., Williams L.L. (2011). Determination of antimicrobial activity of sorrel (*Hibiscus sabdariffa*) on *Escherichia coli* O157:H7 isolated from food, veterinary, and clinical samples. J. Med. Food.

[64] Shan B., Cai Y.Z., Brooks J.D., Corke H. (2007). The in vitro antibacterial activity of dietary spice and medicinal herb extracts. Int. J. Food Microbiol..

[65] Yin M.-C., Cheng W.-S. (2003). Antioxidant and antimicrobial effects of four garlic-derived organosulfur compounds in ground beef. Meat Sci..

[66] Stupar A., Šarić L., Vidović S., Bajić A., Kolarov V., Šarić B. (2022). Antibacterial potential of *Allium ursinum* extract prepared by the green extraction method. Microorganisms.

[67] Li K., Guan G., Zhu J., Wu H., Sun Q. (2019). Antibacterial activity and mechanism of a laccase-catalyzed chitosan–gallic acid derivative against *Escherichia coli* and *Staphylococcus aureus*. Food Control.

[68] Ecevit K., Barros A.A., Silva J.M., Reis R.L. (2022). Preventing microbial infections with natural phenolic compounds. Future Pharmacol..

[69] Putnoky S., Caunii A., Butnariu M. (2013). Study on the stability and antioxidant effect of the *Allium ursinum* watery extract. Chem. Cent. J..

[70] Tomšik A., Šarić L., Bertoni S., Protti M., Albertini B., Mercolini L., Passerini N. (2018). Encapsulations of wild garlic (*Allium ursinum* L.) extract using spray congealing technology. Food Res. Int..

[71] Lachowicz S., Kolniak-Ostek J., Oszmianski J., Wisniewski R. (2016). Comparison of phenolic content and antioxidant capacity of bear garlic (*Allium ursinum* L.) in different maturity stages. J. Food Process. Preserv..

[72] Harris C.S., Mo F., Migahed L., Chepelev L., Haddad P.S., Wright J.S., Bennett S.A.L. (2007). Plant phenolics regulate neoplasic cell growth and survival: A quantitative structure- activity and biochemical analysis. Can. J. Physiol. Pharmacol..

[73] Hiyasat B., Sabha D., Grötzinger K., Kempfert J., Rauwald J.-W., Mohr F.W., Dhein S. (2009). Antiplatelet activity of *Allium ursinum* and *Allium sativum*. Pharmacology.

[74] Jung H.J., Choi J.W., Nam J.H., Park H.J. (2007). Anti-ulcerogenic effects of the flavonoid-rich fraction from the extract of *Orostachys japonicus* in mice. J. Med. Food.

[75] Lien A.P.H., He H., Chuong P.H. (2008). Green tea and health: An overview. J. Food Agric. Environ..

[76] Rietz B., Isensee H., Strobach H., Makdessi S., Jacob R. (1993). Cardioprotective actions of Wild garlic (*Allium ursinum*) in ischemia and reperfusion. Mol. Cell. Biochem..

[77] Asdaq S.M., Inamdar M.N. (2010). Potential of garlic and its active constituent, S-allyl cysteine, as antihypertensive and cardioprotective in presence of captopril. Phytomedicine.

[78] Calderón-Montaño J.M., Burgos-Morón E., Pérez-Guerrero C., López-Lázaro M.A. (2011). Review on the dietary flavonoid kaempferol. Mini Rev. Med. Chem..

[79] Omar S.H., Ramawat K., Mérillon J.M. (2013). Garlic and cardiovascular diseases. Natural Products.

[80] Xu X.Y., Song G.Q., Yu Y.Q., Ma H.Y., Ma L., Jin Y.N. (2013). Apoptosis and G2/M arrest induced by *Allium ursinum* (ramson) watery extract in an AGS gastric cancer cell line. Onco Targets Ther..

[81] Parvu A.E., Parvu M., Vlase L., Miclea P., Mot A.C., Silaghi-Dumitrescu R. (2014). Anti-inflammatory effects of *Alium schoenoprasum* L. leaves. J. Physiol. Pharmacol..

[82] Mihaylova D., Lante A., Tinello F., Krastanov A.I. (2014). Study on the antioxidant and antimicrobial activities of *Allium ursinum* L. pressurized-liquid extract. Nat. Prod. Res..

[83] Finotti E., Di Majo D. (2003). Influence of solvents on the antioxidant property of flavonoids. Food/Nahrung.

[84] Muñiz-Becerá S., Méndez-Lagunas L.L., Rodríguez-Ramírez J., Sandoval-Torres S., López-Ortíz A., Barriada-Bernal L.G. (2022). Modeling of solute transport inside plant tissue during osmotic dehydration of apple. Dry. Technol..

[85] Naveena B.M., Sen A.R., Vaithiyanathan S., Babji Y., Kondaiah N. (2008). Comparative efficacy of pomegranate juice, pomegranate rind powder extract and BHT as antioxidants in cooked chicken patties. Meat Sci..

[86] Jiang J., Zhang X., True A.D., Zhou L., Xiong Y.L. (2013). Inhibition of lipid oxidation and rancidity in precooked pork patties by radical-scavenging licorice (*Glycyrrhiza glabra*) extract. J. Food Sci..

[87] Patinho I., Selani M.M., Saldaña E., Bortoluzzi A.C.T., Rios-Mera J.D., da Silva C.M., Kushida M.M., Contreras-Castillo C.J. (2021). *Agaricus bisporus* mushroom as partial fat replacer improves the sensory quality maintaining the instrumental characteristics of beef burger. Meat Sci..

[88] de Carvalho F.A.L., Lorenzo J.M., Pateiro M., Bermúdez R., Purriños L., Trindade M.A. (2019). Effect of guarana (*Paullinia cupana*) seed and pitanga (*Eugenia uniflora* L.) leaf extracts on lamb burgers with fat replacement by chia oil emulsion during shelf-life storage at 2 °C. Food Res. Int..

[89] Bellucci E.R.B., Munekata P.E.S., Pateiro M., Lorenzo J.M., da Silva Barretto A.C. (2021). Red pitaya extract as natural antioxidant in pork patties with total replacement of animal fat. Meat Sci..

[90] Martins A.J., Lorenzo J.M., Franco D., Vicente A.A., Cunha R.L., Pastrana L.M., Cerqueira M.A. (2019). Omega-3 and Polyunsaturated Fatty Acids-Enriched Hamburgers Using Sterol-Based Oleogels. Eur. J. Lipid Sci. Technol..

[91] Lorenzo J.M., Sineiro J., Amado I.R., Franco D. (2014). Influence of natural extracts on the shelf life of modified atmosphere-packaged pork patties. Meat Sci..

[92] Medyński A., Pospiech E., Kniat R. (2000). Effect of various concentrations of lactic acid and sodium chloride on selected physico-chemical meat traits. Meat Sci..

[93] Manios S.G., Skandamis P.N. (2015). Effect of frozen storage, different thawing methods and cooking processes on the survival of *Salmonella* spp. and *Escherichia coli* O157:H7 in commercially shaped beef patties. Meat Sci..

